# The puzzling issue of silica toxicity: are silanols bridging the gaps between surface states and pathogenicity?

**DOI:** 10.1186/s12989-019-0315-3

**Published:** 2019-08-16

**Authors:** Cristina Pavan, Massimo Delle Piane, Maria Gullo, Francesca Filippi, Bice Fubini, Peter Hoet, Claire J. Horwell, François Huaux, Dominique Lison, Cristina Lo Giudice, Gianmario Martra, Eliseo Montfort, Roel Schins, Marialore Sulpizi, Karsten Wegner, Michelle Wyart-Remy, Christina Ziemann, Francesco Turci

**Affiliations:** 1 UCLouvain, Louvain centre for Toxicology and Applied Pharmacology (LTAP), Brussels, Belgium; 2 Bremen Center for Computational Material Science (BCCMS), Center for Environmental Research and Sustainable Technology (UFT), University of Bremen, Bremen, Germany; 3 INAIL Piemonte, Turin, Italy; 4 G. Scansetti Center, University of Torino, Turin, Italy; 5 Department of Public Health and Primary Care, KU Leuven, Laboratory of Toxicology, Unit of Environment and Health, Leuven, Belgium; 6 Institute of Hazard, Risk and Resilience, Department of Earth Sciences, Durham University, Durham, UK; 7 UCLouvain, Institute of Biomolecular Science and Technology, NanoBioPhysics, Louvain-la-Neuve, Belgium; 8 Department of Chemistry and Nanostructured Interfaces and Surfaces –NIS Centre, University of Torino, Turin, Italy; 9 Instituto de Tecnología Cerámica, Universitat Jaume I, Castellón, Spain; 10 IUF Leibniz Research Institute for Environmental Medicine, Düsseldorf, Germany; 11 Institute of Physics, University of Mainz, Mainz, Germany; 12 ETH Zürich, Zürich, Switzerland; 13 EUROSIL, European Association of industrial silica producers, Brussels, Belgium; 14 Fraunhofer Institute for Toxicology and Experimental Medicine, ITEM, Hannover, Germany; 15 Department of Chemistry, G. Scansetti Center, University of Torino, Turin, Italy

**Keywords:** Silica, Silicosis, Lung cancer, Auto-immune diseases, Surface reactivity, Silanol, Coating, Modelling, Spectroscopy, Atomic force microscopy

## Abstract

**Background:**

Silica continues to represent an intriguing topic of fundamental and applied research across various scientific fields, from geology to physics, chemistry, cell biology, and particle toxicology. The pathogenic activity of silica is variable, depending on the physico-chemical features of the particles. In the last 50 years, crystallinity and capacity to generate free radicals have been recognized as relevant features for silica toxicity. The ‘surface’ also plays an important role in silica toxicity, but this term has often been used in a very general way, without defining which properties of the surface are actually driving toxicity. How the chemical features (e.g., silanols and siloxanes) and configuration of the silica surface can trigger toxic responses remains incompletely understood.

**Main body:**

Recent developments in surface chemistry, cell biology and toxicology provide new avenues to improve our understanding of the molecular mechanisms of the adverse responses to silica particles. New physico-chemical methods can finely characterize and quantify silanols at the surface of silica particles. Advanced computational modelling and atomic force microscopy offer unique opportunities to explore the intimate interactions between silica surface and membrane models or cells. In recent years, interdisciplinary research, using these tools, has built increasing evidence that surface silanols are critical determinants of the interaction between silica particles and biomolecules, membranes, cell systems, or animal models. It also has become clear that silanol configuration, and eventually biological responses, can be affected by impurities within the crystal structure, or coatings covering the particle surface. The discovery of new molecular targets of crystalline as well as amorphous silica particles in the immune system and in epithelial lung cells represents new possible toxicity pathways. Cellular recognition systems that detect specific features of the surface of silica particles have been identified.

**Conclusions:**

Interdisciplinary research bridging surface chemistry to toxicology is progressively solving the puzzling issue of the variable toxicity of silica. Further interdisciplinary research is ongoing to elucidate the intimate mechanisms of silica pathogenicity, to possibly mitigate or reduce surface reactivity.

## Background

Almost 60% of the Earth’s crust is made of silica, mainly in its crystalline form. Crystalline silica (CS) is thus a key industrial product, present in many materials extracted from the ground, and an essential component of numerous products of our daily life, including ceramics, glass, paints, plastics, aids in industrial processes, and in many construction products. Industrial sectors involved with CS include producers or users of sand, gravel, and aggregates, industrial minerals, coal, cement and plaster. Thus, workers are exposed to silica in many occupational settings, and excessive inhalation of respirable CS particles has long been associated with an increased risk of respiratory and systemic diseases. Silicosis is probably the most ancient occupational disease, but obstructive lung diseases, lung cancer or autoimmune diseases are other adverse manifestations related to silica exposure. Effective preventive measures, mainly based on exposure reduction, are available to reduce the occurrence of silica-associated diseases [29]. The recent amendment to Directive 2004/37/EC on the protection of workers from the risks related to exposure to carcinogens or mutagens at work ([1]) fixes a binding limit value (BLV) for respirable CS dust at 0.1 mg/m^3^. Guides and examples of good practice such as those proposed by the European Network on Silica (NEPSI) are recognized as valuable and necessary instruments to complement regulatory measures [37]. The directive revision introduces in its scope “work involving exposure to respirable CS dust generated by a work process”, thus highlighting the importance of material processing, possibly for revealing chemical features critical for the health risks.

The exact mechanism governing the pathogenicity of silica particles remains, indeed, one of the most puzzling issues in toxicology, despite extensive research efforts during the last century (see e.g. [13, 22, 25]). From Hippocrates (400 BC) to the Hawk’s Nest tunnel incident in Gauley Bridge, West Virginia (1927), the most severe diseases associated with CS exposure occurred when particles were generated by cutting, crushing or abrading quartz-containing rocks. At that time, two main intrinsic determinants of toxicity were identified, i.e. crystallinity and fracturing. For some reasons, only crystallinity was taken up and, until the beginning of the past century, silica toxicity has been mainly a subject for occupational physicians. They considered CS particles to be a primary cause of respiratory diseases, without interest in investigating relationships between the way the dust was generated, and its impact on health. In the 1950s–70s, toxicologists began investigating the structure-toxicity relationship with a new approach. King and Wright [27] in the UK and Daniel et al. [11] in France reported modifications of CS with aluminum compounds and their influence on experimental responses to quartz dust. Nagelschmidt [34] pointed out that contact of the quartz surface with minerals, metals or metal salts modifies its toxic potential. In other words, the surface state of quartz was (re-)postulated as a determinant of toxicity. These findings can be regarded as the first steps linking particle toxicology and chemistry.

The need for interdisciplinary collaboration in the search for the structure-toxicity relationship of silica particles became clear and urgent after IARC monograph n° 68 [22]. While identifying the carcinogenicity of CS particles from occupational sources, IARC added a caveat: “*carcinogenicity in humans was not detected in all industrial circumstances studied. Carcinogenicity may be dependent on inherent characteristics of the silica or on external factors affecting its biological activity or distribution of its polymorphs”*. Several publications followed, including “The quartz hazard: A variable entity” [13], “Surface chemistry and quartz hazard” [17], and “Variation of biological responses to different respirable quartz” [7]. Variability thus was recognized as an intrinsic feature of silica toxicity.

In the same period, the need for new and efficient materials prompted the chemical community to develop the production of nanomaterials, including amorphous silica (AS) nanoparticles [31]. As a consequence, surface chemistry, as the clue to the topographic description of particle surfaces, was largely developed. Toxicologists started to use cell culture techniques and the stage was set for large, multidisciplinary collaborations to unveil the interaction of silica particles (crystalline and amorphous) with living matter.

Recent developments in the surface chemistry of silica, cell biology and toxicology have provided new avenues to extend and improve our understanding of the variable reactivity of silica particles. New tools and models are also available to explore the interactions between silica particles and cells. This, collectively, creates a unique momentum for finally elucidating the main mechanisms of silica toxicity. A focused workshop was jointly organized in Torino (September 2018) by the ‘G. Scansetti’ Center of the University of Torino (Italy) and the Louvain centre for Toxicology and Applied Pharmacology (Belgium) to gather both the new and older generations of researchers active in the field, from different horizons and across a range of disciplines, including chemistry, geology, biophysics, biology and toxicology. The aim of the workshop was to discuss recent research on the understanding and prediction of silica toxicity through surface characterization, particle toxicology or preparation of safer materials. This Commentary illustrates how interdisciplinarity can contribute to the understanding of the intimate mechanisms of silica pathogenicity.

### From tools to evaluate the silica surface to the inorganic-bio interface

**Fig. 1:**
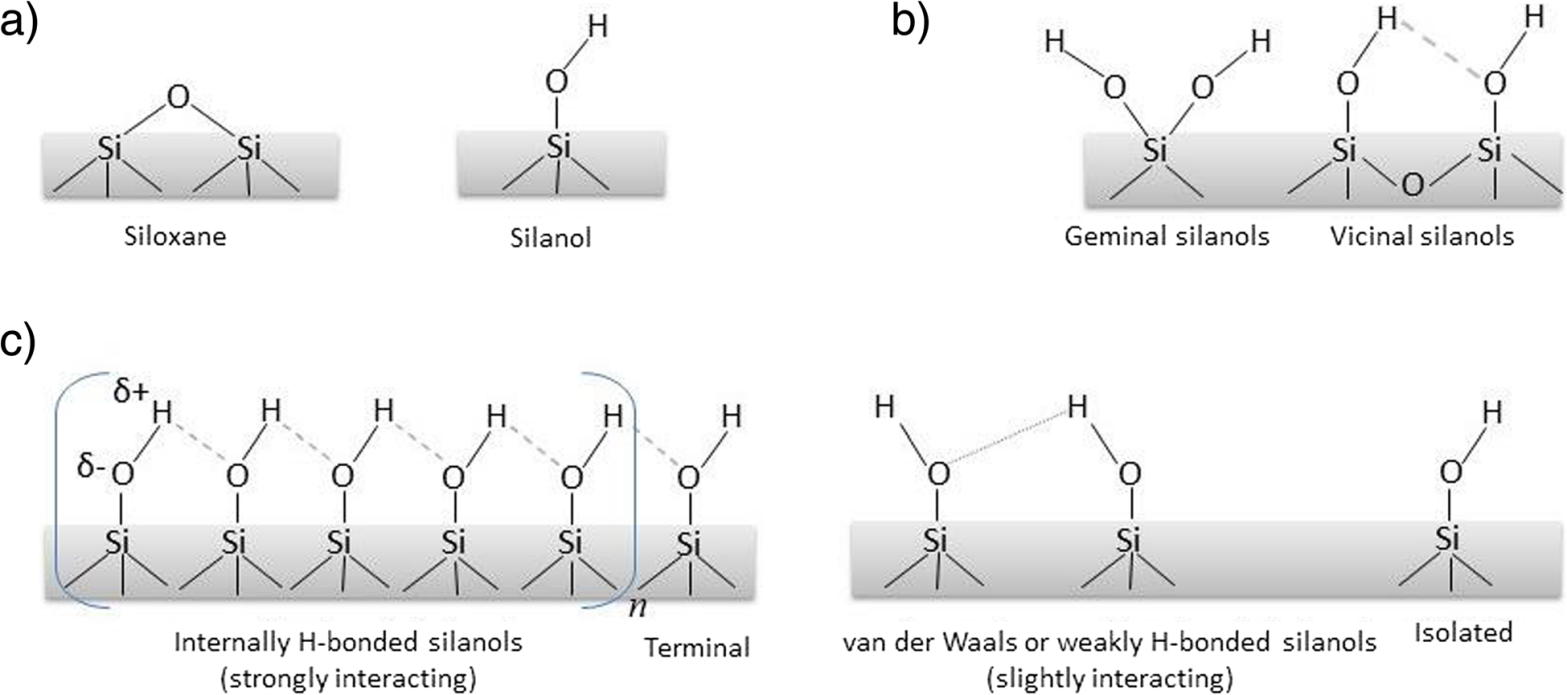
Chemical functionalities on silica surface (**a**). Types of silanols depending on their position on Si atoms (**b**). Types of silanols depending on their mutual distance and bonding (**c**)

The conceptual foundation of research conducted on the surface of silica is that the physico-chemical ‘dialogue’ of silica materials with any medium in contact depends on the relative amount and relative spatial distribution of surface silanols (≡Si-OH; =Si (OH)_2_) and siloxane bridges (≡Si-O-Si≡) (Fig. 1). Strained 3-membered siloxane rings, triggering hydroxyl radical formation, were claimed to have an important role in the toxicity of pyrogenic AS [65], whereas they are considered to be absent at the surface of CS. However, silanol groups also determine a relevant part of the surface of silica, especially in the absence of surface centers active in radical chemistry. Modern physico-chemical methods are now available to finely characterize and quantify silanols at the surface of silica particles, and advanced computational modelling and atomic force microscopy contribute to clarify the intimate interactions between silica surface and biological macromolecules, membrane models or cells.

Surface silanols can be detected and quantified by several complementary methods, including thermal gravimetric analysis, titration, zeta (ζ)-potential, magnetic resonance, and vibrational spectroscopies. While infrared and Raman spectroscopy alone can provide a qualitative picture of the silanol groups present in silica samples, they fail at quantification, unless they are combined with additional measures such as H/D isotopic exchange (see below). Careful *thermogravimetric analysis***,** coupled with mass-spectroscopic analysis of evolved gas, allows determination of the total (internal and surface) silanol content. *Titration* of hydroxyl groups on the particle surface with appropriate reagents such as lithium aluminium hydride (lithium alanate) yields the amount of surface silanols. Not all surface silanol groups are, however, accessible due to steric hindrance. A prerequisite to every quantitative analysis is, therefore, a thorough sample pre-treatment, removing physisorbed water at a temperature of 140–150 °C and application of a vacuum. Such a characterization procedure is illustrated by the example of pyrogenic AS, produced by flame spray pyrolysis at different flame enthalpies, resulting in a range of flame temperatures and particle synthesis times [54]. Thereby, AS nanoparticles with the same specific surface area, but distinct surface chemistry, could be prepared. Surface silanol content of silica synthesised in relatively short and ‘cold’ flames was very high (up to ~ 8 OH/nm^2^), indicating a fully hydroxylated surface. This value was reduced down to ~ 4 OH/nm^2^ for silica made in long and ‘hot’ flames. Moreover, ‘cold-flame’ silica exhibited a higher proportion of hydrogen-bonded vs. isolated silanols and more physisorbed water than ‘hot-flame’ silica of similar average primary particle diameter. Interestingly, the above surface chemistry differences translate to lower in vitro cytotoxic activity in human monocytes for ‘cold-flame’ silica, compared to the ‘hot-flame’ sample of the same specific surface area but with lower surface silanol density [54].

Recent experimental and modeling advances also revealed silanol arrangements, exposed by CS particles, using *infrared (IR) spectroscopy*. IR spectroscopy is widely and easily applicable, and the O-H stretching frequency vibrations (νOH) are highly sensitive to inter-silanols interactions, which depend on the distance between ≡Si-OH groups [8]. Of note, the local arrangement of silanols is known (or supposed) to control important features of the surface behavior of silicas. Thus, a proper collection and analysis of IR νOH signals of surface silanols is critical for a precise, and quantitative, knowledge of silanol families, differing by their inter-silanol distances. A detailed study by IR spectroscopy in a controlled atmosphere, augmented by H/D isotopic exchange, has recently been carried out on highly pure synthetic and natural quartz powders, which were inactive in the release of radical species. The availability of modeling data [32, 33] allowed the assignment of the various νOH sub-bands to H-bonded silanols on different types of surface facets, in good agreement with the crystal habit of particles observed by field emission scanning electron microscopy. No isolated silanols (i.e. more than 6 Å apart) were detected, whereas the main difference in the νOH pattern between the two powders was the much higher relative intensity of a signal assignable to slightly-interacting silanols in natural quartz (Fig. 1). This specific component was found to contribute also to the νOH pattern of pyrogenic AS.

Ab initio *molecular dynamics* simulations can also be used to explore the structure and reactivity of different silica surfaces, including CS and AS. Ab initio simulations allow simulating interfaces, including electronic structure aspects as well as dynamics, and finite temperature effects, which are essential to describe realistic conditions. The acidity of different types of silanols depends on the local environment, also including electrolyte solutions [45]. With ab initio simulations, two types of silanols with different acidity can be identified on fully hydroxylated quartz in contact with water, namely less acid silanols (forming in-plane hydrogen bonds), which exhibit a p*K*_a_ around 8.5, and more acid ones, forming out-of-plane hydrogen bonds, and exhibiting and acidity constant around 5 [55]. When moving from the crystalline to the amorphous surface, a variety of different acidity constants can be observed, which depends not only on the silanol type, but also strongly on the local environment [44]. In more realistic conditions, such as environmental or biological conditions, the silica surface is never in contact with pure water, but, most commonly, it is found in contact with electrolyte solutions. It is, therefore, a crucial question to investigate how properties, such as acidity constants, are modified by the presence of ions. Interestingly, the p*K*_a_’s of the surface silanols follow a combination of the cationic and anionic Hofmeister series in the order p*K*_a_ (neat solution) < p*K*_a_ (NaCl solution) < p*K*_a_ (NaI solution) < p*K*_a_ (KCl solutions) [45] which is in agreement with experimental measurements from Second Harmonic Generation. A rational behind such a ranking can be obtained looking at the microscopic local solvation of the protonated silanols and their conjugated bases, the silanolates SiO^−^. The change in the p*K*_a_ is the result of both water destructuring by alkali halides, as well as of the specific cation/SiO^−^ interaction, depending on the electrolyte [45]. Additionally, for the understanding of surface reactivity, it is also important to discuss how molecular properties, such as acid dissociation constants, may change upon molecule adsorption at the silica/water interface. As shown by a substantial amount of literature, acids at the water surface tend to be ‘less’ acid, meaning that their associated form is favored over the conjugated base. The question is what happens instead at the solid/liquid interface and, in particular, at the solid/liquid interface of interest here, namely the silica/water interface. Probing molecular properties at a buried interface is more difficult than at the water/air interface, however. Therefore, computational predictions may be quite useful and bring new insights. Using a free energy perturbation approach, in combination with electronic structure-based molecular dynamics simulations, it can be shown that, at the quartz/water interface, the acidity of pyruvic acid (a small acid of interest e.g. in atmospheric chemistry processes) is increased by almost two units [38]. Such increased acidity is the result of the specific microsolvation of the molecules at the interface and, in particular, of the stabilization of the deprotonated molecule by the silanols on the quartz surface and the special interfacial water layer [38].

Molecular dynamics simulations are also a valuable tool to investigate interactions at the biotic/abiotic interface. Despite the increase in available computational resources, these methods still suffer from a debilitating timescale problem that greatly reduces the number of phenomena that can be investigated, i.e. properly targeting free energy. So-called ‘enhanced’ sampling methods have been introduced to alleviate this problem [60], and have reached enough maturity to be used for investigation of the complex interface between silica and the biological world. Recent results on the effects of silica nanoclusters of various size and features on membrane models of different composition shed light on the determinants of particle toxicity [12]. Simulations provided a first atomistic picture of the interactions taking place between silica and the membrane of cells, gaining a quantification of the energetics of this process, depending on silica cluster size, membrane composition and cholesterol content. This revealed that silica nanoclusters are highly hydrated, hydrophilic objects that must overcome high barriers to cross the water-lipid interface already at nuclearities of a few atoms. It was observed that there are both local and global destabilizing effects on the membrane structure, upon insertion of the nanoclusters in the membranes. The former appears as a disordering on the lipid tails, within a few nanometers from the particles. The global destabilizing effects appear as water-filled holes deforming the entire membrane sheet. These holes, if confirmed for larger nanoparticles, could be linked to possible pathways of toxicity, based on local perforation and uncontrolled permeabilization of the cell membrane.

*Atomic force microscopy (AFM)* is another approach to elucidate interactions between the biological environment and solid materials such as silica particles. AFM is, to date, one of the most popular techniques to characterize the biophysics of biological interactions [4]. From its invention as an instrument capable of imaging surface topography with atomic level resolution, the technique evolved quickly into a multifunctional toolbox that allows the combination of topographical information with single molecule and single cell force spectroscopy biophysical studies [4]. The translation of AFM-based biophysical approaches, and of their combination with microfluidics and fluorescence imaging, to the study of nanobio-interactions, enables biophysical information to be acquired, such as kinetics and thermodynamics parameters, in physiological conditions and at a single nanoparticle level [18]. Single-molecule and single-cell AFM-based spectroscopy are ongoing to probe the biophysics of interactions between silica particles and scavenger receptors, largely present on cell membranes. This class of receptors, highly expressed in tissue-resident macrophages, interacts with both CS and AS particles. Elucidating the detailed mechanisms of these biomolecular interactions may help explain and, ultimately, prevent some of the toxicological effects of silica, such as inflammasome activation (see below). Dynamic force spectroscopy experiments performed with AFM cantilevers functionalized with silica nanoparticles and scavenger receptor A1 as a proof-of-concept were used to elucidate the specificity of silica-scavenger receptors interactions on model surfaces, resulting in the quantification of biophysical parameters such as the kinetic rate of bond dissociation, and in situ on living cells. In another application, Fluid-FM technology, an instrument combining the force control of an AFM with a microfluidic system, was applied to study the interaction between biological moieties immobilized on nanomaterials and cell machinery on living cells. An analogue approach can be used to study the interactions of quartz particles and phospholipid membrane models, offering new perspectives for the biophysical quantification of the membranolytic potential of silica in biologically relevant conditions. Although still at a preliminary stage, these innovative approaches have the potential of leading nanobio-interactions to unprecedented levels of biological, chemical and physical characterization, with foreseeable benefits in several fields, such as (nano) toxicology, nanomedicine and material science.

### From the inorganic-bio interface to toxicological responses

A significant bridge between surface chemistry and toxicology has been established by recent studies examining the relationship between silanols and pathogenic responses to silica particles. Particle toxicology studies often start with basic membranolytic tests, using red blood cell membrane damage (haemolysis) as a proxy for more complex mechanisms of toxicity. Quartz is highly haemolytic and often used as a positive control in haemolysis tests. Early investigations on the membranolytic potential of quartz focused on a systematic analysis of the physico-chemical properties of silica particles relevant for membranolysis [42]. These studies concluded that surface features of the particles definitely have a greater impact on membranolysis than structural properties. Indeed, besides quartz, AS particles such as vitreous silica particles and the pyrogenic nano AOX50® are also highly membranolytic, whereas synthetic quartz crystals of respirable size exposing as-grown intact crystal faces [39] are not membranolytic [58]. As particles unable to generate hydroxyl or carboxylate radicals were, in some cases, highly membranolytic (e.g. AOX50®), mediators of membrane damage other than silica-derived reactive oxygen species (ROS) were considered. Upon specific thermal treatments affecting the silanol distribution, the membranolytic activity of both AS and CS particles was reduced, suggesting a major role for silanols in the interaction with cell membrane moieties.

Next, membranolysis of phagolysosomes was identified as an early key event promoting activation of the inflammasome in macrophages and epithelial cells [48, 50]. This enzymatic machinery was discovered to trigger and sustain the inflammatory reaction caused by silica particles [10, 14, 19], a key process in the development of silicosis, lung cancer and autoimmunity [6, 46]. Membranolytic activity of a set of selected quartz particles was correlated with their capacity to activate the NOD-like receptor family, pyrin domain-containing 3 (NLRP3) inflammasome and to trigger a pro-inflammatory response in vitro [41], suggesting that silanols may be implicated in the labilisation of the phagolysosomal membrane and the inflammatory reaction.

A further piece to the puzzle was added by using respirable as-grown quartz crystals, obtained via an innovative hydrothermal synthesis procedure [39, 58]. Unlike the quartz dusts previously used in experimental studies, which were mostly of mineral origin and therefore ground to obtain fine powders forming conchoidal fractures on the surface, synthetic quartz crystals have regular shapes and native intact surfaces, near to an ideal perfect quartz crystal surface as modelled by Musso et al. [32]. As for membranolysis, as-grown quartz crystals with intact surfaces were not cytotoxic to lung cells in vitro, confirming that the activity of quartz particles is not necessarily contingent to crystallinity [40]. After fracturing the surface of as-grown quartz crystals by mechanical grinding, their biological activity was markedly increased. Formation of surface defects and a higher heterogeneity of silanol populations were identified as the causes of this increased reactivity of the fractured quartz surface [58]. Efforts now aim to validate, in vivo, the relevance of specific populations of reactive silanols, and to set up assays for predicting the respiratory hazard of silica particles, based on the analysis of their surface silanol distribution. The role of silica surface ageing after fracturing in biological responses would also represent another important aspect to investigate in relation to silanol stability over time. It should, however, be noted that, while fracturing appears important for silica particles to create specific silanol populations which drive membranolysis and inflammasome activation, it may not be essential for other inflammasome-activating crystals such as monosodium urate (MSU) or cholesterol crystals. The surface chemistry and crystal structures of silica and MSU are very different, but they have in common surface groups, able to form strong H-bonds with membrane phospholipids [63]. While, for silica, fracturing is a critical step to introduce defects and a relative amount/specific distribution (i.e. orientation and position) of H-bonding silanols not present on fully hydroxylated surfaces, for MSU crystals the surface functionalities might be present at the native surface, or at the acidic pH of inflamed tissue or phagolysosome, without a need for fracturing. The mechanism of inflammasome activation by MSU might also depend on other pathways than lysosome labilisation, e.g. lipid sorting and Syk activation at the cell membrane level, or protein adsorption (see e.g. Shi et al. [53]). The picture is less clear for cholesterol crystals [56].

The presence of impurities within the crystalline structure of silica particles is another determinant of the variable pathogenic activity of silica [13, 22], especially for crystalline polymorphs with an open lattice structure such as cristobalite. Recent work on cristobalite has explored, how these impurities can interfere with surface chemistry and toxicity. In cristobalite-rich dusts, substitutions of cations, such as Al, for Si in the open cristobalite structure, and impurities occluding the silica surface, have been hypothesised as mechanisms which might dampen toxicity [20, 36]. Cristobalite in both volcanic ash and calcined diatomaceous earth contains these structural substitutions (with several wt.% Al + Na observed in cristobalite in both dust types) [20, 36]. Cristobalite is usually also partially occluded by Al-rich glass and other components from the original volcanic lava, diatomaceous frustules, and from other sediments. Through occlusion, the surface area of cristobalite available for reactivity with cellular components, is substantially reduced. Aluminium has previously been shown to play a role in the amelioration of CS toxicity, with previous work using aluminium lactate as a coating almost entirely dampening the toxicity of quartz (see e.g. [5, 15]). To test the hypothesis that structural substitutions affect particle toxicity, cristobalite was synthesised and doped with incremental quantities of Na and/or Al [35]. Synthetic analogues were necessary because the natural samples contained accessory minerals, which made it impossible to isolate the effect of structural substitutions [35]. The ability of the samples to induce cytotoxicity and pro-inflammatory responses was assessed in vitro in macrophages, and in red blood cells (haemolysis). Doping reduced both cytotoxicity to macrophages and haemolytic capacity. Al-only doping was also more effective at decreasing cristobalite reactivity than Al + Na co-doping [35]. The reduced reactivity of doped cristobalite can be attributed to both structural impurities and a lower abundance of CS in doped samples, caused by progressive crystallisation of accessory phases, such as albite, as Al and Na reached saturation. Both impurities and occlusion, therefore, can reduce the toxic potential of cristobalite and may help to explain the low reactivity of some cristobalite-rich dusts, such as volcanic ash. Related to surface chemistry, the presence of Al and Na atoms at the particle surface likely also interferes with the distribution and quantity of active silanols and their acidity. Whilst further work is required to determine if these toxicological effects translate to altered pathogenicity, the results have potential implications for the regulation of silica exposures within the cristobalite industry, if some types of ‘impure’ CS could be proven to be less pathogenic than purer forms.

### From toxicological effects back to surface inactivation

As the physico-chemical determinants of silica toxicity appear linked to surface silanol groups, chemical blocking of these reactive groups might represent a strategy to render silica less toxic, and to increase workers’ safety. As already mentioned, several approaches were considered to modify, mask or inactivate the surface of CS particles with different compounds, including dipalmitoyl lecithin [62], Al lactate [5, 15] or organosilanes [59].

Several studies explored the role of the silica surface in the toxicity of the reference CS sample, DQ12, modified with polyvinyl-pyridine-N-oxide (PVNO) or Al lactate. In an in vivo rodent model, these surface modifications reduced the ability of DQ12 to induce a persistent pulmonary inflammation, DNA damage in alveolar epithelial cells and lung tissue remodelling, including fibrosis, whereas particle clearance from the lung was increased [2, 3, 28, 43]. The inhibitory effects were generally stronger for PVNO than for the Al lactate modification. Complementary in vitro investigations with macrophages and lung epithelial cell lines provided more insight into the underlying cellular and molecular mechanisms. For instance, in line with the in vivo observation on lung clearance, the uptake of particles by macrophages in vitro was higher for surface-modified DQ12, whereas the uptake by epithelial cells was higher for the pristine material [2, 52]. Surface modification of DQ12 also inhibited its ability to activate the pro-inflammatory Nuclear Factor kappa B (NFκB) signalling pathway in macrophages as well as in epithelial cells. Further investigation suggests that the activation of the canonical NFκB pathway in the epithelial cells predominantly proceeds in an indirect manner, through mediators released from DQ12-activated macrophages rather than through direct interactions between the particles and epithelial cells [61]. Recent in vitro and in vivo investigations using pristine versus PVNO-modified DQ12, also revealed the importance of the quartz surface for the activation of the NLRP3 inflammasome [43]. As such, this provided further support for the importance of this signal pathway in quartz-induced lung inflammation and tissue remodelling [14, 19].

There are currently concrete efforts to bring preventive CS surface coating into industrial application. Two EU projects have developed and implemented, at an industrial scale, cost-effective coating technologies, based on stable, covalent masking of surface silanol groups to inhibit CS toxicity [59, 64]. Both wet [16, 66] and dry coating methods [30] have recently been reported. In contrast to known approaches to dampen toxicity with substances such as Al lactate, which act by ionic interaction with silanol groups, these surface-coating technologies are based on stable, covalent bonds between the coating agent (e.g. the organosilane Dynasylan® SIVO 160) and the reactive surface silanols, to reduce toxicity in a more stable manner. The most challenging issues were to define appropriate treatment parameters (reaction time, dosage, additive selection, catalyst, etc.) and to specify physico-chemical tests for cheap and fast determination of coating effectiveness during the development phase. In this regard, measurement of the ζ-potential was found to be extremely sensitive, and correlated well with toxicological results [24]. For implementation of such coating approaches at the industrial scale, costs of the treatment and the technical behaviour of the coated silica in the industrial application represent additional critical issues. The technical behaviour of the wet-coated silica was successfully tested at industrial scale in several ceramic companies producing tiles, sanitary ware and tableware goods. Furthermore, the dry coating method has so far been tested in a more multi-sectorial study (glass, pigment, adhesive, elastomer and foundry producers) at pilot plant scale. The preliminary technical and toxicological tests suggest that the recently developed dry coating method is very promising. From the toxicological point of view, such development processes need to be guided by biological tests to ensure functionality of the coating methods and coating effectiveness in biological systems. Taking into account the 3Rs principles in animal research, besides in vivo studies, it is also indispensable to have appropriate and validated in vitro screening models and predictive biological endpoints in place. Primary rat alveolar macrophages in short-term culture turned out to represent a sensitive and meaningful in vitro screening model in this regard, with membrane damage and direct DNA damage as the main screening endpoints, and Al lactate as a tool to differentiate between silica-dependent and –independent biological effects. The predictive value of macrophage-based in vitro results was confirmed in a 90-day intratracheal instillation study in rats [66]. In contrast, acellular incubation approaches, using artificial alveolar and lysosomal fluids, with subsequent cell incubations for determination of coating stability, were not able to completely predict the in vivo results. Using in vitro and in vivo screening models and diverse endpoints, it could be demonstrated that some covalent coatings with, e.g. organosilanes, are able to effectively and stably block CS toxicity in the lung for up to 90 days, without interfering with technical process quality in industrial production. Therefore, such coating strategies represent a promising tool to render CS handling safer.

### Towards new targets for silica toxicity

Recent research on the interaction between silica particles and cell receptors has revealed that AS nanoparticles affect the function of cellular ionic channels [49]. AS nanoparticles are known to affect the airway epithelium [31], but the molecular targets of these particles remain largely unknown. The observation that AS nanoparticles interact with the plasma membrane and affect the barrier function of the epithelium initiated new research lines. Transient Receptor Potential (TRP) channels are cation-permeable channels that regulate epithelial barrier function. Out of all TRP tested (TRPA1, TRPV1, TRPV4, TRPM3 and TRPM8), the TRP Vanilloid 4 (TRPV4) has been shown to be strongly affected by AS nanoparticles, with a significant decrease of its activation by the powerful synthetic agonist GSK1016790A. Ludox® particles (a commonly available commercial 9 nm AS particle) inhibit the activation of the TRPV4 channel in mouse and human airway epithelial cells, as well as in a heterologous system expressing the mouse isoform of this channel. Patch-clamp current recordings showed a direct inhibition of the channel activity, while functional measurements demonstrated that AS nanoparticles abrogate the increase of ciliary beat frequency, triggered by activation of TRPV4. The inhibition of TRPV4 by AS nanoparticles occurs at concentrations and time scales much smaller than those reported for other effects of these particles. Taken together, the cation channel TRPV4 is an immediate and sensitive molecular target, through which AS nanoparticles may impair the clearance function of ciliated cells, potentially resulting in defective defensive responses of the airway epithelium. Future investigations may need to assess the role of the silica surface in inhibiting this important channel.

Innate immune system recognition is also a sophisticated mechanism which promptly recognizes silica particles and engages crescendo immune and tissue responses. Major progress has been achieved in recent years regarding recognition of microorganisms by the innate immunity, notably by integrating a set of distinct receptors designated pattern recognition receptors (PRRs), which serve as sensors for monitoring the extracellular and intracellular compartments for microbial residues. After infection, this elaborate system also detects debris from dying cells (known as danger-associated molecular patterns, DAMP) and perturbations in cytoplasmic homeostasis (recently defined as homeostasis-altering molecular processes, HAMP). Decades ago, such a PRR-mediated sensing system did not appear plausible for silica particles because they were considered different from biological structures such as bacterial cell-wall components or viral nucleic acids. The discovery that scavenger receptors (SR, a subfamily of PRR) sense silica particles in macrophages [23] shifted the opinion of researchers in particle toxicology and suggested that innate immunity can specifically recognize silica particles and initiate biological responses to these particles. Recent developments in silica particle sensing demonstrated that the silica-recognition systems also comprise inflammasome machinery (PRR) [9], alarmin release (DAMP) [47] and membrane destabilization (HAMP) [26]. Recent studies elegantly clarified the mechanisms underlying the direct recognition of silica particles by SR. Negatively charged silica particles (both CS and AS) directly interact with a conserved motif of SR containing positively charged amino acids. The silica/receptor binding consequently activates specific signaling pathways, resulting in the production of TNF-α and IL-1 family members, which coordinate early responses to silica [57]. In 2008, three distinct reports concurrently revealed a new PRR-related intracellular sensing axis, comprising NLRPs, which is pivotal in silica recognition and IL-1 activation after phagocytosis (reviewed in [48]). Silica-induced dying cells and cell death pathways also have an important role in the initiation of tissue responses against silica particles. The release of necrotic cell or apoptotic body contents after membrane rupture (membranolysis) acts as a danger signal to initiate rapid immune responses. Molecules generated by dying cells include the alarmin IL-1α that accounts for the upstream immunological cues regulating innate immunity and initiating tissue responses to silica [47]. A simple contact between the macrophage plasma membrane and silica particles is also sufficient to trigger TNF-α production in the absence of phagocytosis. It has been suggested that radicals generated at the surface of silica causes membrane lipid peroxidation, extracellular Ca^2+^ influx, and TNF-α release, which occurs within the first minutes of cell exposure to silica [51]. However, little is known about the role of the surface functionalities (silanols) in triggering early recognition responses. These emerging recognition systems survey the extracellular or cytosolic spaces for detecting silica particles or particle-related cell signatures, and operate in a collective manner to promote cytokine release and tissue responses [21]. The progressive development of fibrosis, cancer, infection, and autoimmune diseases after silica exposure appears when particles constantly activate PRR-mediated particle recognition, induce persistent cytokine release and promote long-lasting immune responses. These unforeseen aspects of silica-sensing processes by the innate immune system have shaken up our knowledge of early host responses against silica particles. Thus, exploring the collective actions of the PRR pathways sensing silica particles opens new horizons to decipher the mechanisms of silica toxicity.

## Conclusions

The workshop illustrated how the puzzling issue of the variable toxicity of silica can be progressively unravelled by interdisciplinary research bridging surface chemistry to toxicology. It highlighted several key aspects that will fuel further interdisciplinary research for the elucidation of the intimate mechanisms of silica pathogenicity for possible mitigation or reduction of surface reactivity, and hence prevention of adverse health effects:

- new physico-chemical methods can finely characterize and quantify silanols at the surface of silica particles;
- computational modelling is unravelling some of the molecular mechanisms behind the interactions between silanols and biomolecules or cellular membranes;
- force microscopy with ad hoc functionalized tips offers unique opportunities to explore the interactions between cells and the surface of silica particles;
- fracturing of silica particles induces a perturbation of the regular crystalline face, generating, upon contact with atmospheric components, specific silanol populations (slightly-interacting and isolated silanols), which impart membranolytic and inflammatory activity to the respirable CS particles;
- impurities at the surface of some CS (cristobalite), including substitution of Al and/or Na and occlusion of particle surfaces by Al-rich accessory minerals, likely influences the H-bonding potential of silanols at the particle surface and can reduce toxicity;
- the toxicity of CS particles can effectively be reduced by surface coating processes masking silanol functionalities, also at the industrial level;
- the discovery of new molecular targets of silica particles (crystalline and amorphous) in the immune system and in epithelial lung cells allows exploration of new toxicity and surface-driven pathways for these particles.

## Data Availability

N**/a**

## Abbreviations

AFM: Atomic force microscopy
AS: Amorphous silica
CS: Crystalline silica
DAMP: Danger associated molecular pattern
HAMP: Homeostasis altered molecular process
IARC: International Agency for Research on Cancer
NLRP: Nucleotide-binding oligomerization domain, Leucine rich Repeat and Pyrin domain containing
PRR: Pathogen recognition receptor
ROS: Reactive oxygen species
SR: Scavenger receptor
TRP: Transient receptor potential
